# Impacts of people at-risk of either cow milk allergies or lactose intolerance on their daily calcium intake and bone mineral density

**DOI:** 10.3389/fnut.2024.1421275

**Published:** 2024-08-16

**Authors:** Kornkanok Kongpharm, Parinda Nakklay, Chunhakan Kongtong, Pichaya Tanapumchai, Lukkamol Prapkree, Narisa Rueangsri, Alongkote Singhato

**Affiliations:** ^1^Nutrition and Dietetics Division, Faculty of Allied Health Sciences, Burapha University, Chonburi, Thailand; ^2^Sodexo at the University of Kansas Health System, Olathe, KS, United States

**Keywords:** bone mineral density, calcium intake, cow milk allergy, DEXA, lactose intolerance

## Abstract

**Background:**

People who are at risk of either cow milk allergies or lactose intolerance may need to avoid consuming milk and milk products, which are well-known abundant sources of calcium (Ca). This limitation in calcium intake could affect bone health. Currently, there is limited knowledge on the impact of avoiding the consumption of milk and milk products on the daily Ca intake and bone mineral density (BMD) of people at risk of cow milk allergies. This study aimed to investigate the differences between the amount of Ca intake and BMD values between people who are at risk of cow milk allergies and those who are not.

**Methods:**

A total of 80 participants were recruited, and further divided into two groups, the at-risk cow milk allergies (AR) group (*n* = 40) and the normal (NOR) group, using the cow milk allergies and lactose intolerance screening questionnaire. The anthropometric assessment, body composition analyses, 3-day dietary record, and bone mass density (wrist and ankle bones) measurement of all participants were collected using the dual x-ray absorptiometry (DEXA) technique to compare the differences of variables between the two groups.

**Results:**

The participants in the AR group presented a significantly lower amount of Ca intake (317 mg/day) than those in the NOR group (623 mg/day) (*p* < 0.05). The bone mineral density (BMD) parameters indicated that the NOR group presented significantly higher T-scores and BMD values of the wrist (T-score = −0.27 and BMD = 0.57 g/cm2) and ankle (T-score = −0.01 and BMD = 0.59 g/cm2) bones when compared with the AR group (T-score = −1.96 and BMD = 0.48 g/cm2 for the wrist bone, and T-score = −1.18 and BMD = 0.47 g/cm2 for the ankle bone) (*p* < 0.05). In addition, the results indicated significantly positive correlations between the amount of Ca intake and the T-scores and BMD values of both the wrist and ankle bones among all participants (*p* < 0.05).

**Conclusion:**

In this responding sample, participants at risk of cow milk allergies experienced a significantly negative impact on the amount of Ca intake and BMD values. Professionals in nutrition and dietetics should provide nutrition education and strategies that can enhance the Ca intake among this population to help them meet the daily Ca intake recommendation, ultimately leading to better bone health.

## Introduction

It is well known that cow’s milk is an abundant source of calcium (Ca) and protein with high biological value, both of which are essential for human growth and development (1, 2). In many countries, inadequate Ca intake has been reported due to low consumption of milk and milk products such as yogurts and cookies. For example, more than 95% of Chinese adults consumed less than 800 mg/day of calcium, which is below the Dietary Recommended Intake (DRI) (3). The average Ca intake in Japan and Korea is approximately 500 mg per day or less (4). A recent report from Iran revealed that milk consumption declined from 26.77 kg *per capita* in 1991 to 22.68 kg *per capita* in 2021 (5). Another cohort study in China found that the median (P25 and P75) intake of milk and its products among Chinese adults was 20.5 g/day (with the 25th and 75th percentiles at 5.19 g/day and 75.0 g/day, respectively) from 1999 to 2010. This is much lower than in European and North American nations, where the intake is 368.4 ± 282.5 g/day (6). In Thailand, a small amount of Ca consumption of 300 mg per day was reported in Thai adults (7). Long-term inadequate Ca intake leads to bone-related complications, such as osteopenia, osteoporosis, and bone fracture (8, 9). Therefore, inadequate calcium consumption is one of the primary nutritional and public health concerns contributing to low bone mineral density (BMD), which may adversely affect the quality of life (10). Therefore, adequate milk consumption should be encouraged across all age groups to ensure adequate Ca intake according to their DRI.

There is a high prevalence of milk allergies and lactose intolerance (affecting between 1 and 3% of the population), which influences people’s dietary habits by causing them to avoid milk and milk products (11). The diagnosis of milk allergies can be performed through the oral food challenge and skin prick methods to determine the condition of milk allergies (12, 13), whereas lactose intolerance can be tested by conducting the hydrogen breath test (14). However, undiagnosed people could be considered at risk of cow milk allergies or lactose intolerance if they suffer from symptoms, such as skin rashes, breathing difficulty, diarrhea, bloating, vomiting, and anaphylaxis, after consuming milk or milk products (15). Hence, people at risk of cow milk allergies or lactose intolerance should avoid milk and milk products to minimize and prevent the occurrence of adverse symptoms (16). According to previous studies, inadequate Ca intake, especially from milk and milk products, could cause low BMD, which contributes to bone health problems. Thus, individuals with cow milk allergies may have a higher risk of Ca deficiency compared to those without the allergy, as they may avoid milk and milk products, which potentially affects their BMD values. This study, therefore, aimed to investigate the impact of cow milk allergies on the amount of daily Ca intake, biochemical parameters related to bone health, and BMD values.

## Methods

### Questionnaires used in this study

The cow milk allergies and lactose intolerance screening questionnaire, which was developed and adapted from a previous study (17), was used to screen participants in this study for the risk of cow milk allergies and lactose intolerance. The list of closed-ended questions that appeared in the questionnaire was based on the symptoms of cow milk allergies and lactose intolerance, such as breathing difficulty, diarrhea, skin rash, and vomiting, among others (18). Participants were asked to complete all “Yes or No” questions to identify their risk for cow milk allergies. Examples of questions include “You have had nausea every time after intake of milk or milk products such as ice cream, cookies, yogurts, milk tablet, etc.” and “You have had diarrhea every time after intake of milk or milk products such as ice cream, cookies, yogurts, milk tablet, etc.” If participants responded “Yes” to any of these questions, they were considered at risk of cow milk allergies.

The other two questionnaires used in this study were a baseline questionnaire to collect general characteristics data (body mass index (BMI), daily length of sun exposure, length of weekly exercise, etc.) and a 3-day food record questionnaire to collect dietary habits data of participants on 2 week days and 1 weekend day. All questionnaires were reviewed, proofread, and validated by three experts in nutrition and dietetics using the index of item-objective congruence (IOC) method before submitting to the ethical review board of Burapha University for approval (approval no. IRB-063/2566).

### BMD and body composition measurement

The participants’ BMD values were determined using the dual x-ray absorptiometry (DEXA) technique (Osteo checker, Ampall Co Ltd., Seoul, South Korea). Daily calibration was performed every time before launching to measure participants’ BMD values. T-scores and BMD values (g/cm^2^) of the wrist and ankle bones of all participants were determined and recorded. For body composition analysis, total body water, muscle weight, body fat mass, and resting energy expenditure (REE), among others, were analyzed using the bioelectrical impedance analysis (BIA) technique (InBody270, InBody Inc., Seoul, South Korea).

### Study participants

After enrollment, quota sampling was performed to divide the participants into two groups, the at-risk group (*n* = 40) and the normal group (*n* = 40), using the developed cow milk allergies and lactose intolerance screening questionnaire. The participants who responded as having any symptoms of cow milk allergies or lactose intolerance in the screening questionnaire were enrolled in the at-risk group, whereas the participants who responded as having no symptoms were included the control group. The participants were included until the number of participants in each group reached the target. Furthermore, all 80 participants were recruited for this study based on the following inclusion criteria: Thai adults aged between 18 and 50 years who can read and write Thai and have no medical history of infectious diseases. The exclusion criteria were participants who currently take dietary supplements or herbal supplements, those who have a medical history of mental illness, pregnant or lactating women, and those who currently take any medications. All participants signed the informed consent form before participating in this study.

### Study procedure

**Figure 1:**
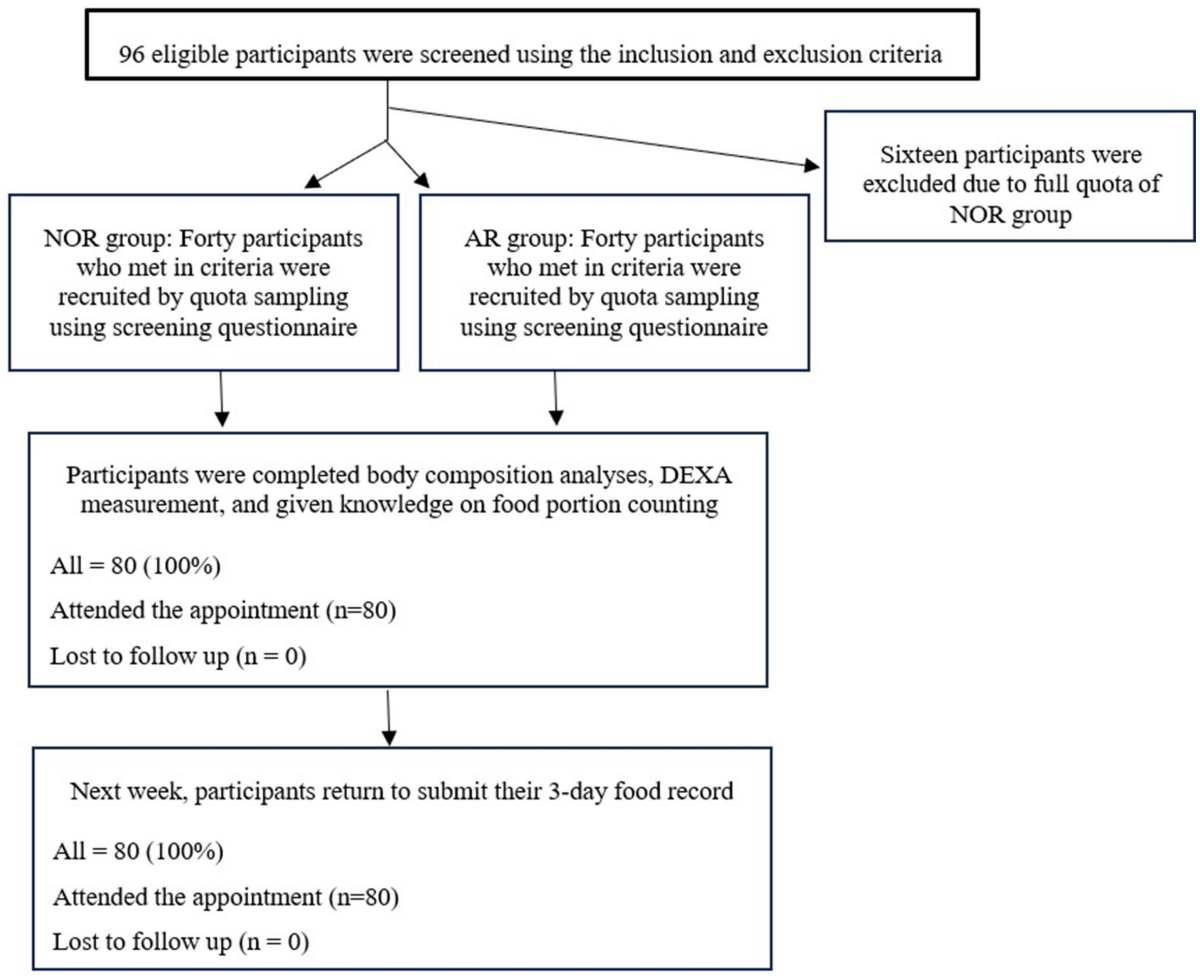
Flowchart of study procedures.

After recruitment, all participants who were interested in participating in this study were appointed and invited to the Nutrition and Dietetics Division, the Faculty of Allied Health Sciences, Burapha University, Chonburi province. All of them completed the cow milk allergies and lactose intolerance screening questionnaire. Participants who responded “Yes” to any questions about the symptoms after the intake of milk or milk products were grouped in the at-risk (AR) group, whereas participants who responded as having no symptoms after the intake of milk or milk products were grouped in the normal group (NOR). This quota sampling method was conducted until the target number of participants was reached in each group. Then, all participants completed the baseline questionnaire and the anthropometric assessment. Furthermore, participants’ BMD values were measured using the DEXA and their body composition was analyzed using the BIA technique. In addition, all participants were provided with knowledge on food portion counting and asked to record their food intake on 2 weekdays and 1 weekend day in the given 3-day food record questionnaire provided. All participants were asked to return the food record questionnaire in the next 1 week for further calculating the nutrient intake using the nutritional software package, INMUCAL-nutrients (version 4.0), developed by the Institute of Nutrition, Mahidol University, Thailand (Figure 1).

### Statistical analyses

General information of all participants, such as age, BMI, length of weekly exercise, and length of daily sun exposure, along with the data on nutrient intake, and BMD values, was represented as mean ± SD. The independent paired *t*-test was conducted to compare the mean differences between the two groups. Pearson’s chi-squared test was conducted to compare the differences in the percentage data, and the Pearson correlation coefficient was used to analyze the correlation between the amount of Ca intake and bone health condition. All statistical analyses were performed using the statistical software package Predictive Analytics Software Statistics (SPSS Inc., Chicago, Il), version 24.0. A statistically significant difference was set at a *p*-value of <0.05.

## Results

### Participants’ characteristics and body composition

There were no significant differences in sex, age, and BMI between the two groups. However, the participants in the NOR group had a daily length of sun exposure of 96 min per day, which was significantly higher than the 59 min per day observed in the AR group (*p* < 0.05). For body composition analysis, the results revealed that the body muscle of the participants was 10.97 kg in the NOR group and 9.50 kg in the AR group, while the body fat mass of the participants was 23.62 kg in the NOR group and 27.27 kg in the AR group. Both parameters—body muscle and body fat mass— of the participants in the NOR group were significantly different from those in the AR group (*p* < 0.05). In addition, no significant differences were found in the total body water, weight of minerals, fat-free mass, waist–hip ratio, and REE between the two groups (Table 1).

**Table 1:** General characteristics and baseline anthropometric data of participants.

Variables	NOR group (*n* = 40)	AR group (*n* = 40)	*p*-value
Sex			
Male, *n* (%)	18 (45.00)	20 (50.00)	0.65
Female, *n* (%)	22 (55.00)	20 (50.00)	
Age, mean (SD)	24.20 (6.27)	23.67 (5.68)	0.69
BMI, mean (SD)	21.51 (4.28)	22.49 (5.07)	0.35
Sun exposure duration a day (minute), mean (SD)	96.25 (65.97)	59.00 (60.94)	<0.05*
Length of daily exercise (minute), mean (SD)	70.62 (102.89)	51.62 (41.85)	0.28
Total body water (L), mean (SD)	28.67 (3.64)	29.77 (3.15)	0.15
Body muscle (kg), mean (SD)	10.97 (2.04)	9.50 (1.73)	<0.05*
Body fat mass (kg), mean (SD)	23.62 (3.62)	27.27 (3.29)	<0.05*
Mineral (kg), mean (SD)	2.90 (0.35)	2.78 (0.44)	0.17
Fat-free mass (kg), mean (SD)	35.07 (1.30)	35.75 (2.23)	0.10
Waist–hip ratio, mean (SD)	0.79 (0.13)	0.74 (0.12)	0.08
REE (kcal), mean (SD)	1,257.23 (119.48)	1,223.33 (121.64)	0.21

*Significant difference at *p* < 0.05.

### The energy and nutrient intake of participants

The energy distribution and Ca intake of participants in both groups were calculated. There was no significant difference between total energy and energy distribution percentage from carbohydrate intake between both groups. However, the dietary habits data indicated that the participants in the NOR group obtained energy distribution from protein at 19.05%, which was significantly higher than that obtained by the participants in the AR group, who obtained energy distribution from protein at 11.82% (*p* < 0.05). In addition, the participants in the NOR group obtained energy distribution from fat at 27.25%, which was significantly lower than that obtained by the participants in the AR group, who obtained energy distribution from fat at 34.45% (*p* < 0.05). Furthermore, the amount of Ca intake of the participants in the NOR group was 623.01 mg per day, which was significantly higher than the 317.43 mg per day intake recorded for the AR group participants (*p* < 0.05) (Table 2).

**Table 2:** Data on the dietary habits of participants.

Variables	NOR group (*n* = 40)	AR group (*n* = 40)	*p*-value
Total energy intake, mean (SD)	1618.81 (524.92)	1693.55 (537.90)	0.53
%kcal from carbohydrate, mean (SD)	53.70 (5.37)	53.97 (7.48)	0.85
%kcal from protein, mean (SD)	19.05 (4.96)	11.82 (2.74)	<0.05*
%kcal from fat, mean (SD)	27.25 (6.37)	34.45 (8.06)	<0.05*
Ca intake (mg), mean (SD)	623.01 (568.28)	317.43 (186.16)	<0.05*

### Participants’ BMD values

The results revealed that the participants in the NOR group obtained a T-score of 0.27 for the wrist bone and − 0.01 for the ankle bone, while the participants in the AR group obtained a T-score of −1.96 for the wrist bone and − 1.18 for the ankle bone. For BMD, the results revealed that the participants in the NOR group obtained a BMD value of 0.57 g/cm2 for the wrist bone and 0.59 g/cm2 for the ankle bone, while the participants in the AR group obtained a BMD value of 0.48 g/cm2 for the wrist bone and 0.47 g/cm2 for the ankle bone. There were significant differences in all T-scores and BMD values of both the wrist and ankle bones between the two groups (*p* < 0.05) (Table 3).

**Table 3:** *T*-scores and BMD values of participants.

*T*-scores and BMD values	NOR group (*n* = 40)	AR group (*n* = 40)	*p*-value
*T*-score of the wrist bone, mean (SD)	−0.27 (1.37)	−1.96 (0.54)	<0.05*
BMD value of the wrist bone (g/cm^2^), mean (SD)	0.57 (0.08)	0.48 (0.04)	<0.05*
*T*-score of the ankle bone, mean (SD)	−0.01 (1.39)	−1.18 (0.62)	<0.05*
BMD value of the ankle bone (g/cm^2^), mean (SD)	0.59 (0.08)	0.47 (0.05)	<0.05*

**Figure 2:**
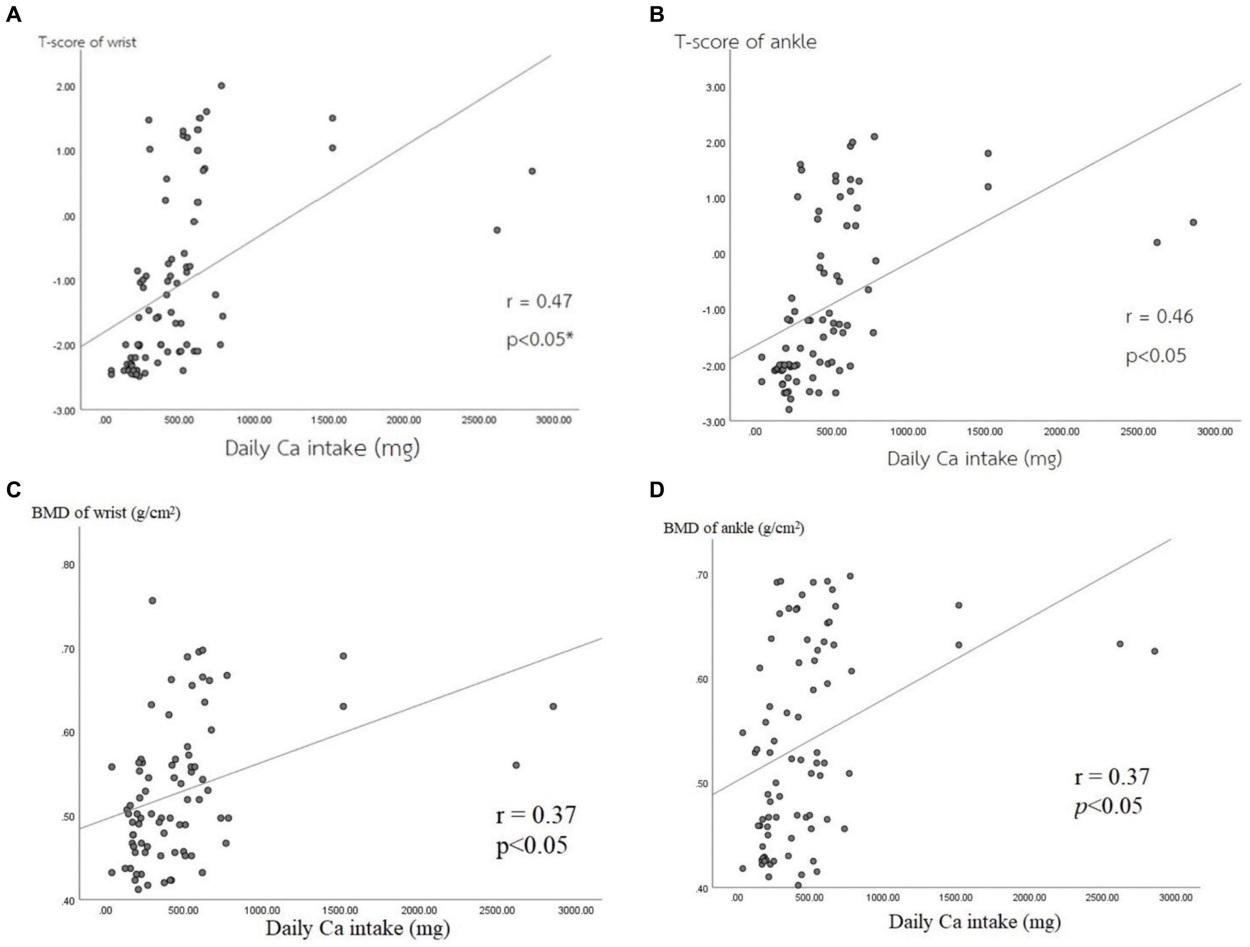
Correlations between bone mass density parameters and Ca intake. **(A)** T-score of wrist, **(B)** T-score of ankle, **(C)** BMD of wrist, **(D)** BMD of ankle.

Moreover, the results indicated a positive correlation between the amount of Ca intake and bone density parameters among all 80 participants. The T-score of the wrist bone had a significantly positive correlation with the amount of Ca intake (*r* = 0.47) (*p* < 0.05) (Figure 2A), and the T-score of the ankle bone also had a positive correlation with the amount of Ca intake (*r* = 0.46) (*p* < 0.05) (Figure 2B). For BMD values, there was a significantly positive correlation between the BMD value of the wrist bone and the amount of Ca intake (*r* = 0.37) among participants (Figure 2C), as well as between the BMD value of the ankle bone and the amount of Ca intake (*r* = 0.37) (*p* < 0.05) (Figure 2D).

## Discussion

This study revealed that participants at risk of cow milk allergies, who have lower T-scores of bones and lower BMD values, might be experiencing these conditions due to a lower intake of Ca, which is the major element in bone formation (19, 20). Moreover, the findings of this study revealed that there were positive correlations between the amount of Ca intake and the bone condition parameters of both the wrist and ankle bones. These findings supported the previous studies that established a correlation between a higher amount of Ca intake and increased BMD values (21, 22). Interestingly, a study pointed out that BMD was significantly improved with a sufficient amount of Ca intake of above 566 mg/day (23). However, neither group met the recommended daily Ca intake for adults, which is advised to be 900 mg/day (24). The results of this study are in line with the findings of previous studies on insufficient Ca intake among populations in various countries (25–27). Hence, it is necessary for individuals at risk of cow milk allergies to incorporate other food sources of Ca, such as green leafy vegetables, hard tofu, small fish, and Ca-fortified products (28), to help them minimize the risk of developing bone complications. In addition, the length of sun exposure of participants may have been one of the factors that affected the bone condition parameters due to its role in promoting vitamin D (25-hydroxyvitamin-D [25(OH)D]) synthesis, which enhances Ca absorption (29). Although there was a significant difference between the groups regarding the length of sun exposure, the length of sun exposure in both groups was close to the recommended length (1 h) that is required to maintain the optimum 25(OH)D level pointed out by a previous study conducted in South Asia (30). The body compositions of the participants in the NOR group and AR group showed differences in terms of body muscle and body fat mass. This could be attributed to the difference in dietary habits, with the participants in the NOR group having a higher energy distribution from protein than those in the AR group. It is possible that the AR group avoided consuming milk and milk products, which are rich sources of high-biological value protein (31). In contrast, although the participants in the NOR group obtained lower energy from dietary fat than those in the AR group, they had higher body fat than those in the AR group. The possible effects of different physical activities and the length of weekly exercise of the participants in their daily lives fell short of the recommended exercise time (150–300 min of moderate exercise/week) (32).

Avoidance of milk and milk products, which are rich sources of Ca, poses a major challenge for people at risk of cow milk allergies and lactose intolerance (33, 34). The mechanism of cow milk protein allergy is caused by an abnormal immune response to cow milk proteins such as casein and beta-lactoglobulin, which is commonly an IgE-mediated response (35). The release of histamine and inflammatory cytokines leads to symptoms, such as breathing difficulty, skin rash, and anaphylaxis (36). The skin prick test and the oral food challenge are examples of diagnosis methods to determine the conditions of cow milk protein allergy, which must be conducted under the supervision of experts (37). Therefore, alternative sources of Ca intake, such as green leafy vegetables, hard tofu, or even goat milk, are recommended for the nutritional management of people with cow milk protein allergies. These alternatives are suggested because they contain lower levels of alphaS1-casein compared to cow’s milk, assisting in meeting adequate Ca intake (38). Lactose intolerance involves a different mechanism compared to cow milk protein allergy. It is caused by an abnormality in the gastrointestinal system that results in a deficiency of lactase, the enzyme secreted from brush border functioning in the breakdown of lactose in milk (39). For diagnosing lactose intolerance, the hydrogen breath test is commonly used to measure hydrogen gas levels in the exhaled breath, resulting from undigested lactose fermentation in the intestine (40). Hence, the nutritional management for people with lactose intolerance involves incorporating other sources of Ca and milk without lactose, such as lactose-free milk products (41). Both cow milk protein allergy and lactose intolerance are reported as the causes of patients’ insufficient Ca intake that may impact their bone health (42). This study was the first in Asia to investigate the impact of cow milk allergies and lactose intolerance on the calcium intake and bone health of at-risk people. One of the limitations of the study is that the participants were not medically diagnosed with either cow milk allergies or lactose intolerance. Therefore, future studies are required to investigate people who are diagnosed with cow milk protein allergies and lactose intolerance as different conditions need different approaches and distinct nutritional management. Another limitation is the use of quota sampling and the small sample size, which may limit the generalizability to a wider population. In addition, future studies are needed to collect biochemical parameters related to bone health, such as serum Ca, alkaline phosphatase, and parathyroid hormone (43). In conclusion, people at risk of either cow milk allergies or lactose intolerance face negative impacts on their Ca intake and bone health.

## Data Availability

The raw data supporting the conclusions of this article will be made available by the authors, without undue reservation.

## References

[1] Zhang X Chen X Xu Y Yang J Du L Li K. Milk consumption and multiple health outcomes: umbrella review of systematic reviews and meta-analyses in humans. Nutr Metab (Lond). (2021) 18:7. doi: 10.1186/s12986-020-00527-y, PMID: 33413488 PMC7789627

[2] Woźniak D Cichy W Dobrzyńska M Przysławski J Drzymała-Czyż S. Reasonableness of enriching Cow's Milk with vitamins and minerals. Food Secur. (2022) 11:1079. doi: 10.3390/foods11081079, PMID: 35454665 PMC9025252

[3] Huang K Fang H Yu D Guo Q Xu X Ju L. Usual intake of micronutrients and prevalence of inadequate intake among Chinese adults: Data from CNHS 2015-2017. Nutrients. (2022) 14:4714. doi: 10.3390/nu14224714 36432400 PMC9696081

[4] Ohta H Uenishi K Shiraki M. Recent nutritional trends of calcium and vitamin D in East Asia. Osteoporos Sarcopenia. (2016) 2:208–13. doi: 10.1016/j.afos.2016.08.002, PMID: 30775488 PMC6372740

[5] Roustaee R Eini-Zinab H Ghodsi D Mehrparvar Hosseini E Omidvar N Hosseini H. A 30-year trend of dairy consumption and its determinants among income groups in Iranian households. Front Public Health. (2024) 12:1261293. doi: 10.3389/fpubh.2024.1261293, PMID: 38425466 PMC10903262

[6] Dehghan M Mente A Rangarajan S Sheridan P Mohan V Iqbal R. Association of dairy intake with cardiovascular disease and mortality in 21 countries from five continents (PURE): a prospective cohort study. Lancet. (2018) 392:2288–97. doi: 10.1016/s0140-6736(18)31812-9 30217460

[7] Balk EM Adam GP Langberg VN Earley A Clark P Ebeling PR. Global dietary calcium intake among adults: a systematic review. Osteoporos Int. (2017) 28:3315–24. doi: 10.1007/s00198-017-4230-x, PMID: 29026938 PMC5684325

[8] Voulgaridou G Papadopoulou SK Detopoulou P Tsoumana D Giaginis C Kondyli FS. Vitamin D and calcium in osteoporosis, and the role of bone turnover markers: a narrative review of recent data from RCTs. Diseases. (2023) 11:29. doi: 10.3390/diseases11010029, PMID: 36810543 PMC9944083

[9] Shlisky J Mandlik R Askari S Abrams S Belizan JM Bourassa MW. Calcium deficiency worldwide: prevalence of inadequate intakes and associated health outcomes. Ann N Y Acad Sci. (2022) 1512:10–28. doi: 10.1111/nyas.14758, PMID: 35247225 PMC9311836

[10] Rizzo M Tammaro G Guarino A Basso M Cozzolino A Mariconda M. Quality of life in osteoporotic patients. Orthop Rev (Pavia). (2022) 14:38562. doi: 10.52965/001c.38562, PMID: 36267218 PMC9568431

[11] Flom JD Sicherer SH. Epidemiology of Cow's Milk allergy. Nutrients. (2019) 11:5. doi: 10.3390/nu11051051, PMID: 31083388 PMC6566637

[12] Bukhari E Gabrielli S McCusker C Upton J Grunebaum E Chan ES. Skin prick test in milk allergic patients undergoing oral immunotherapy: does the milk form used for skin tests matter? Front Allergy. (2022) 3:974626. doi: 10.3389/falgy.2022.974626, PMID: 36003413 PMC9393482

[13] Calvani M Bianchi A Reginelli C Peresso M Testa A. Oral food challenge. Medicina (Kaunas). (2019) 55. doi: 10.3390/medicina55100651, PMID: 31569825 PMC6843825

[14] Usai-Satta P Oppia F Lai M Cabras F. Hydrogen breath tests: are they really useful in the nutritional Management of Digestive Disease? Nutrients. (2021) 13:974. doi: 10.3390/nu13030974, PMID: 33802839 PMC8002624

[15] Vandenplas Y Bajerova K Dupont C Eigenmann P Kuitunen M Meyer R. The Cow's Milk related symptom score: the 2022 update. Nutrients. (2022) 14:2682. doi: 10.3390/nu14132682, PMID: 35807862 PMC9268587

[16] Giannetti A Toschi Vespasiani G Ricci G Miniaci A di Palmo E. Cow's Milk protein allergy as a model of food allergies. Nutrients. (2021) 13:1525. doi: 10.3390/nu13051525 33946553 PMC8147250

[17] Saad K Elgenidy A Atef M Abdelsattar MK Al-Ashwah M Hammad EM. Cow's Milk-related symptom score for cow's milk allergy assessment: a meta-analysis for test accuracy. Pediatr Res. (2023) 93:772–9. doi: 10.1038/s41390-022-02334-y, PMID: 36253506

[18] Vandenplas Y Marchand J Meyns L. Symptoms, diagnosis, and treatment of Cow's Milk allergy. Curr Pediatr Rev. (2015) 11:293–7. doi: 10.2174/1573396311666150731113059 26239112

[19] Bailey RL Zou P Wallace TC McCabe GP Craig BA Jun S. Calcium supplement use is associated with less bone mineral density loss, but does not lessen the risk of bone fracture across the menopause transition: data from the study of Women's health across the nation. JBMR Plus. (2020) 4:e10246. doi: 10.1002/jbm4.10246, PMID: 31956850 PMC6957983

[20] Méndez-Sánchez L Clark P Winzenberg TM Tugwell P Correa-Burrows P Costello R. Calcium and vitamin D for increasing bone mineral density in premenopausal women. Cochrane Database Syst Rev. (2023) 2023:CD012664. doi: 10.1002/14651858.CD012664.pub2, PMID: 36705288 PMC9881395

[21] Liu Y Le S Liu Y Jiang H Ruan B Huang Y. The effect of calcium supplementation in people under 35 years old: a systematic review and meta-analysis of randomized controlled trials. eLife. (2022) 11:11. doi: 10.7554/eLife.79002, PMID: 36164828 PMC9514846

[22] Vannucci L Fossi C Quattrini S Guasti L Pampaloni B Gronchi G. Calcium intake in bone health: a focus on calcium-rich mineral waters. Nutrients. (2018) 10:1930. doi: 10.3390/nu10121930, PMID: 30563174 PMC6316542

[23] Fang A Li K Li H Guo M He J Shen X. Low habitual dietary calcium and linear growth from adolescence to young adulthood: results from the China health and nutrition survey. Sci Rep. (2017) 7:9111. doi: 10.1038/s41598-017-08943-6, PMID: 28831091 PMC5567300

[24] Cormick G Belizán JM. Calcium intake and health. Nutrients. (2019) 11:7. doi: 10.3390/nu11071606, PMID: 31311164 PMC6683260

[25] Rana ZH Bourassa MW Gomes F Khadilkar A Mandlik R Owino V. Calcium status assessment at the population level: candidate approaches and challenges. Ann N Y Acad Sci. (2022) 1517:93–106. doi: 10.1111/nyas.14886 36044378 PMC9804725

[26] Cairoli E Aresta C Giovanelli L Eller-Vainicher C Migliaccio S Giannini S. Dietary calcium intake in a cohort of individuals evaluated for low bone mineral density: a multicenter Italian study. Aging Clin Exp Res. (2021) 33:3223–35. doi: 10.1007/s40520-021-01856-5, PMID: 33909280 PMC8668846

[27] Guo X Gao J Meng X Wang J Zhang Z Song Q. Association of Dietary Calcium Intake with Bone Health and Chronic Diseases: two prospective cohort studies in China. Front Nutr. (2021) 8:683918. doi: 10.3389/fnut.2021.683918, PMID: 35004796 PMC8740131

[28] Coppola S Carucci L Oglio F Di Sarra C Ozen G Berni CR. Nutritional strategies for the prevention and Management of Cow's Milk allergy in the pediatric age. Nutrients. (2023) 15:3328. doi: 10.3390/nu15153328, PMID: 37571266 PMC10421120

[29] Fleet JC. Vitamin D-mediated regulation of intestinal calcium absorption. Nutrients. (2022) 14:3351. doi: 10.3390/nu14163351, PMID: 36014856 PMC9416674

[30] Patwardhan VG Mughal ZM Chiplonkar SA Webb AR Kift R Khadilkar VV. Duration of casual sunlight exposure necessary for adequate vitamin D status in Indian men. Indian J Endocrinol Metab. (2018) 22:249–55. doi: 10.4103/ijem.IJEM_473_17, PMID: 29911040 PMC5972483

[31] Auestad N Layman DK. Dairy bioactive proteins and peptides: a narrative review. Nutr Rev. (2021) 79:36–47. doi: 10.1093/nutrit/nuab097, PMID: 34879145 PMC8653944

[32] Bull FC Al-Ansari SS Biddle S Borodulin K Buman MP Cardon G. World Health Organization 2020 guidelines on physical activity and sedentary behaviour. Br J Sports Med. (2020) 54:1451–62. doi: 10.1136/bjsports-2020-102955, PMID: 33239350 PMC7719906

[33] Vandenplas Y Al-Hussaini B Al-Mannaei K Al-Sunaid A Helmi Ayesh W El-Degeir M. Prevention of allergic sensitization and treatment of Cow's Milk protein allergy in early life: the middle-east step-down consensus. Nutrients. (2019) 11:1444. doi: 10.3390/nu11071444, PMID: 31248015 PMC6683055

[34] Facioni MS Raspini B Pivari F Dogliotti E Cena H. Nutritional management of lactose intolerance: the importance of diet and food labelling. J Transl Med. (2020) 18:260. doi: 10.1186/s12967-020-02429-2, PMID: 32590986 PMC7318541

[35] Tang R Lyu X Liu Y Zhu M Yang X Wu Z. Four clinical phenotypes of cow's milk protein allergy based on dairy product specific IgE antibody types in North China. Front Immunol. (2022) 13:949629. doi: 10.3389/fimmu.2022.949629, PMID: 36275773 PMC9585381

[36] Weimer DS Demory BM. Underlying immune mechanisms involved in Cow's Milk-induced hypersensitivity reactions manifesting as atopic dermatitis. Cureus. (2022) 14:e27604. doi: 10.7759/cureus.27604, PMID: 36059314 PMC9433788

[37] Jensen SA Fiocchi A Baars T Jordakieva G Nowak-Wegrzyn A Pali-Schöll I. Diagnosis and rationale for action against Cow's Milk allergy (DRACMA) guidelines update—III—Cow's milk allergens and mechanisms triggering immune activation. World Allergy Organ J. (2022) 15:100668. doi: 10.1016/j.waojou.2022.100668, PMID: 36185551 PMC9483786

[38] AL QH Al-Saadi JS Al-Rikabi AKJ Altemimi AB Hesarinejad MA Abedelmaksoud TG. Exploring the health benefits and functional properties of goat milk proteins. Food Sci Nutr. (2023) 11:5641–56. doi: 10.1002/fsn3.3531, PMID: 37823128 PMC10563692

[39] Misselwitz B Butter M Verbeke K Fox MR. Update on lactose malabsorption and intolerance: pathogenesis, diagnosis and clinical management. Gut. (2019) 68:2080–91. doi: 10.1136/gutjnl-2019-318404, PMID: 31427404 PMC6839734

[40] Robles L Priefer R. Lactose intolerance: what your breath can tell you. Diagnostics (Basel). (2020) 10:412. doi: 10.3390/diagnostics10060412, PMID: 32560312 PMC7344825

[41] Li A Zheng J Han X Yang S Cheng S Zhao J. Advances in low-lactose/lactose-free dairy products and their production. Food Secur. (2023) 12:2553. doi: 10.3390/foods12132553, PMID: 37444291 PMC10340681

[42] Goldberg MR Nachshon L Sinai T Epstein-Rigbi N Oren Y Eisenberg E. Risk factors for reduced bone mineral density measurements in milk-allergic patients. Pediatr Allergy Immunol. (2018) 29:850–6. doi: 10.1111/pai.12972, PMID: 30099766

[43] Ladang A Rousselle O Huyghebaert L Bekaert AC Kovacs S Le Goff C. Parathormone, bone alkaline phosphatase and 25-hydroxyvitamin D status in a large cohort of 1200 children and teenagers. Acta Clin Belg. (2022) 77:4–9. doi: 10.1080/17843286.2020.1769285, PMID: 32441564

